# Population genetics of neotropical *Culex quinquefasciatus* (Diptera: *Culicidae*)

**DOI:** 10.1186/s13071-014-0468-8

**Published:** 2014-10-05

**Authors:** André Barretto Bruno Wilke, Paloma Oliveira Vidal, Lincoln Suesdek, Mauro Toledo Marrelli

**Affiliations:** Departamento de Epidemiologia, Faculdade de Saúde Pública, Universidade de São Paulo, São Paulo, SP Brasil; Laboratório Parasitologia, Instituto Butantan, São Paulo, SP Brasil; Programa de Pós-Graduação em Biologia da Relação Patógeno-Hospedeiro, Instituto de Ciências Biomédicas, Universidade de São Paulo, São Paulo, SP Brasil; Programa de Pós-Graduação em Medicina Tropical, Instituto de Medicina Tropical de São Paulo, Universidade de São Paulo, São Paulo, SP Brasil

## Abstract

**Background:**

*Culex quinquefasciatus* mosquitoes can be found in almost every major city of Brazil and are vectors of filariasis and several arboviruses. Microsatellite markers have been widely used to uncover the genetic structure of various groups of insect populations. The aim of this study was to glimpse the genetic structure of *Cx. quinquefasciatus* in Brazil.

**Methods:**

Nine populations were sampled across Brazil (one of them from a laboratory colony - COL) and another one from Argentina and process regarding the variability of six microsatellite *loci*.

**Results:**

The analyzed *loci* revealed moderate population genetic structure (mean F_st_ = 0.12). Dendrograms of genetic distances evidenced two major population clusters, respectively corresponding to the northern and southern populations. The hybrid population *Cx. pipiens/quinquefasciatus* (from La Plata, Argentina) and the colony population fell outside the major clusters. Those clusters were substructured and there was a significant correlation between genetic and geographic distances and environmental variables (r = 0.51; p > 0.001 and r = 0.46; p > 0.004).

**Conclusions:**

Multilocus cluster Bayesian analysis confirmed that populations are mutually distinct, and the set of results point to genetic differences among populations. The presumable low gene flow among them may be due to the large geographic distances (>1000 km) and to the environmental heterogeneity of the sampled areas. The genetic structure observed in this study may lead to the best understanding of *Cx. quinquefasciatus* demographical diversity as well as their genetic variations patterns in Brazil so far unknown.

## Background

*Culex pipiens* species complex sharing morphological similarities can be found in urban areas and are responsible for the transmission of several pathogens [1,2]. One member of this complex, *Culex quinquefasciatus*, is adapted to live in tropical and subtropical areas while *Cx. pipiens* mosquitoes lives in temperate regions [3]. *Cx. quinquefasciatus* is well established in Brazil and can be found in almost all major cities [4]. This mosquito transmits lymphatic filariasis caused by *Wuchereria bancrofti* (Spirurida: Onchocercidae), most cases occur in tropical regions of the planet, 800 million people live in endemic areas and 120 million people are infected [5]. There is active transmission of filariasis in the state of Pernambuco, Brazil [5,6]. This species can also transmit several arboviruses such as West Nile Virus and Saint Louis encephalitis [7-11].

*Cx. quinquefasciatus* mosquitoes are able to survive in polluted waters where there are no natural predators, which leads to excessive growth of the population [12]. This phenomena may be due to phenotypic plasticity and might have a role in environmental adaptation and insecticide resistance with implications that made chemical interventions no longer effective for the control of mosquito populations [13-22]. Therefore, a better knowledge of the genetic structure of insect populations is required for the development of effective strategies for vector control. Molecular markers have been widely used at the resolution of taxonomic studies and population genetics issues of several insect groups [23-27].

Microsatellites were utilized to seek for population variations in *Cx. quinquefasciatus* mosquitoes on Hawaii that are associated with landscape altitude variations which lead to population structures caused by spatial interactions among vector, host and parasite. Disease patterns are interconnected with elevation gradient structuration and therefore there are epidemiologically important outcomes [28]. Hybrid *Cx. quinquefasciatus*/*pipiens* mosquitoes can be found in Uruguay, northern Argentina and western of Brazil while *Cx. pipiens* can be found in the southern regions of Latin America [29].

Morais *et al.* [30] found that Brazilian populations’ of *Cx. quinquefasciatus* from tropical regions had wing shapes distinct from subtropical populations, sorting out the northern populations (tropical zone) of southern populations (sub-tropical zone), also demonstrated by the use of ace-2 molecular marker. This event can be explained by greater gene flow among populations of the same region than between regions, indicating a barrier, yet to be confirmed [30].

It is still not well known how *Cx. quinquefasciatus* mosquitoes are demographically distributed, in addition to the paucity of information on their genetic variations. Recent studies indicate that this species varies regionally and thus different control approaches are needed [29,30]. A better understanding of the mosquito population genetic structure might be useful to anticipate vector borne disease distribution patterns and play a decisive role in epidemiological interventions [31]. Herein, we seek to estimate the genetic diversity of *Cx. quinquefasciatus* populations in Brazil by analyzing ten populations from several regions with distinct climatic and geographical characteristics.

## Methods

### Collection of specimen - mosquito samples

**Figure 1:**
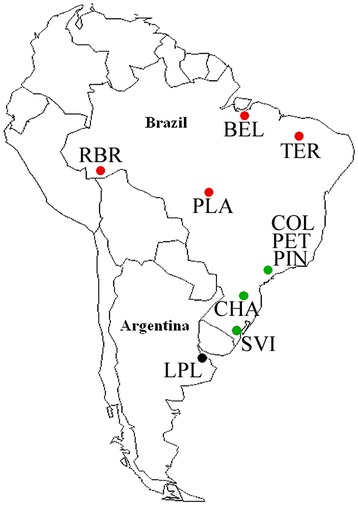
**Map of South America showing mosquito population capture sites (n = 10).** Coloured dots indicate microsatellite-based genetic clustering of populations.

Adult *Cx. quinquefasciatus* mosquitoes were captured from ten sampling localities (Table 1, Figure 1) using a battery powered aspiration device near breeding sites during February and March (rain season) in 2008 [32]. Each captured mosquito was identified by taxonomic keys and stored in silica until processed for DNA extraction [2,30], they were then processed with taxon-specific PCR primers, *ace-2 locus* to distinguish and characterize populations and zones of hybridization [30]

**Table 1:** **Mosquito populations, collecting sites and geographic coordinates**

**Population**	**Origin**	**Coordinates**
RBR	Rio Branco - AC	10°1′17.47″S/67°46′36.43″W
BEL	Belém - PA	1°34′30.83″S/48°27′58.97″W
COL	São Paulo - SP	23°34′0.44″S/46°43′57.61″W
LPL	La Plata - Argentina	34°55′2.86″S/57°56′57.13″W
TER	Teresina - PI	5°6′22.28″S/42°46′28.18″W
PLA	Pontes e Lacerda - MT	15°15′42.29″S/59°17′59.74″W
SVI	Santa Vitória - RS	33°30′33.51″S/53°19′16.71″W
CHA	Chapecó - SC	27°3′23.01″S/52°35′9.32″W
PET	São Paulo - SP	23°29′1.07″S/46°30′14.41″W
PIN	São Paulo - SP	23°38′48.31″S/46°43′37.07″W

### Populations

All mosquito populations were collected from urban areas with different climate and urbanization characteristics. RBR’ mosquito inhabit an equatorial region with temperatures ranging between 25–40°C, located in the Amazon region. The BEL population is located in the banks of the Amazon River, with only 6.5% of home sewage connected to the collection network resulting in an abundance of breeding sites. COL is the laboratory colony, highly monomorphic as a result of high levels of inbreeding due to isolation in the laboratory since 1980. LPL population comprises of hybrid mosquitoes from a hybridization zone between *Culex quinquefasciatus/pipiens* in La Plata, Argentina. The TER population resides in a semi humid tropical area, located in the transition zone between the semi-arid northeast of Brazil and the Amazon rainforest, as well as PLA located in a transition zone between Cerrado and the Amazon rainforest. SVI is located in the extreme south of Brazil, and has a temperate climate. CHA is located in the Uruguay River basin with an annual average temperature of 19.60°C. PET mosquitoes were collected in a linear park with 12.5 million m^2^ located within the city of São Paulo. PIN population was collected in a highly urbanized area, subject to selective pressures caused by humans and is highly anthropophilic.

### DNA extraction and microsatellite amplifications

Thirty adult females of each population were used to perform the genomic DNA extraction, according to the DNeasy Blood and Tissue kit (Qiagen, California, USA). Six microsatellite *primers* previously utilized in *Cx. quinquefasciatus* were selected (Table 2) [31,33]. These six primers, named: CA-115 - GT-14 - GT-108 - GA-12 - CA-118 - ATG-09, were fluorescent-labeled (FAM - HEX - NED) and tested in our *Cx. quinquefasciatus* populations.

**Table 2:** **Selected** ***Culex quinquefasciatus primers***

**Primers**	**Sequence 5′-3′**	**Allele Size (bp)**	**Number of Alleles**
**CxqA115**	F: GTCGTCAAACTGCCAATAA	86-296	30
	R: GCGGAAATAGAACAAACG		
**CxqGT14**	F: TGTTAGCCTAGTGGGAAGGTG	106-200	18
	R: AATCCACCATGCACGGATAC		
**CxqGT108**	F: CGTGTTTTATAGGCTTCTTTC	106-316	27
	R: TCTTCCTTAACTTTACCCACTC		
**CxqGA12**	F: ACCCGTTCTGGCAACACTG	110-188	17
	R: TGGTGCGGATGGACGTT		
**CxqA118**	F: ACCCCGAGCCAACCTTAT	112-254	26
	R: CCCCCATTTCACACCTGT		
**CxqATG9**	F:CCACTCAAACTAAAACACCACA	108-300	26
	R: AATGCCATAACCATCGTCAT		

Amplification reactions (PCR) were performed as in Edillo *et al.* [31] and Smith *et al.* [33], in an AG-22331 Thermocycler Eppendorf (Hamburg Germany). After the PCR amplification multiplex dilutions were performed using three fluorescent-labeled primer (FAM, HEX and NED), where 3 μL of PCR product of each primer was added to 21 μL ultra-pure water for each sample for a final volume of 30 μL. A second dilution was performed with 2 μL of diluted PCR product resuspended in 7.5 μL of Formamide HI-DI (Applied Biosystems, Warrington, UK), 0.5 μL of molecular size standard GeneScan 500 LIZ (Applied Biosystems, Warrington, UK) was added for a final volume of 10 μL. Samples were processed in the ABI 3730 automatic sequencer (Applied Biosystems, Foster, CA, USA).

### Fragment size determination and statistical analyses

Fragment sizes were determined with GeneMarker software package (Softgenetics). A parametric t-test was performed for each population, aiming to quantify genetic differentiation. Hardy-Weinberg equilibrium deviations and genetic variation indices were obtained utilizing GENEPOP V4.0 (http://genepop.curtin.edu.au/) [34,35]. Multilocus genotypes of each individual were then processed by the program Arlequin [36]; F_st_ values were generated to survey the level of genetic structure between populations. Genetic isolation by geographic distances and pairwise multilocus number of migrants (Nm) between populations per generation were estimated in GENEPOP 4.0 utilizing regression of pairwise F_st_/(1-F_st_) and the geographical distances were shown for a straight-line in kilometers [35]. The selected environmental variables allowed us to examine how environmental factors might be associated with genetic variations of *Cx. quinquefasciatus.* The selected parameters incorporate annual trends and environmental characteristics of collection sites (mean annual temperature, annual precipitation, altitude, inhabitants and Human Development Index) [37]. Correlations between genetic, geographic distances and environmental variables were estimated with Statistica v7.0 software.

The software Structure 2.3.4 [38] was utilized to appoint the amount of genetic clusters enclosed into the data. To define the value of K, from 1–12 twelve runs were made using default settings, the formula ΔK = m ([L”K])/s [L (K)] was applied [39].

## Results

### Genetic diversity

Hardy-Weinberg Equilibrium Test was performed for all six microsatellite *loci*. It was observed after Bonferroni correction [40] that heterozygosity was lower than expected in 36 of 60 tests that could be conducted (p < 0.00083) and the mean F_is_ value was 0.34. Out of the 150 possible tests, significant linkage disequilibrium was revealed only between *locus* ATG-09 and GT-14 in the RBR sample suggesting the *loci* were not in linkage (Table 3).

**Table 3:** **Six microsatellite** ***loci*** **in** ***Culex quinquefasciatus*** **mosquitoes’ genetic diversity**

**Locus**	**Population**	**No. of alleles**	**Observed heterozygosity (%)**	**Expected heterozygosity (%)**	**Fis**	**P**
GT-108	ACR	11	50	78	0.3636	**0**
	BEL	13	63.3	87	0.2764	**0.0003**
	COL	3	36.7	39.7	0.0754	0.0203
	PLA	11	66.7	88.7	0.2521	**0**
	TER	12	56.7	83.7	0.327	**0.0019**
	PLA	11	56.7	88	0.3614	**0**
	SVI	9	63.3	80.7	0.2173	0.0622
	CHA	10	50	85.7	0.4141	**0**
	PET	10	70	82	0.1488	0.0778
	PIN	11	50	83.3	0.4049	**0**
ATG-09	ACR	8	60	69.7	0.1414	0.0487
	BEL	10	36.7	70.7	0.4855	**0**
	COL	3	0	12.7	1,000	**0.0003**
	PLA	14	60	85.7	0.304	**0**
	TER	7	46.7	69.7	0.335	**0.006**
	PLA	9	76.7	73	−0.0529	0.1283
	SVI	8	76.7	84	0.0907	0.1593
	CHA	14	50	86.7	0.4284	**0**
	PET	8	86.7	79	−0.0983	**0**
	PIN	12	70	83	0.16	0.0296
CA-118	ACR	6	36.7	53	0.311	0.0245
	BEL	13	53.3	88	0.399	**0**
	COL	3	0	12.7	1,000	**0.0002**
	PLA	7	40	60.3	0.3409	**0**
	TER	6	20	64	0.692	**0**
	PLA	11	40	82.3	0.5177	**0**
	SVI	9	46.7	82	0.4349	**0**
	CHA	11	46.7	77.3	0.4007	**0**
	PET	7	26.7	38.3	0.3085	**0.0027**
	PIN	9	56.7	75.7	0.2536	**0.0003**
GT-14	ACR	10	26.7	71.3	0.6297	**0**
	BEL	13	43.3	74	0.4178	**0**
	COL	/	/	/	/	/
	PLA	5	5.9	48.8	0.8824	**0**
	TER	5	34.5	66.2	0.4834	**0.0004**
	PLA	5	36.7	70.7	0.4863	**0.0003**
	SVI	5	17.8	62.8	0.7202	**0**
	CHA	5	28	70.4	0.6084	**0**
	PET	5	35.7	62.5	0.4334	**0.0002**
	PIN	5	34.5	71.7	0.5246	**0**
GA-12	ACR	6	70	73.7	0.0492	0.502
	BEL	10	70	74.7	0.0624	0.0247
	COL	2	46.7	50.3	0.0794	0.7243
	PLA	4	75.9	61	−0.2457	**0.0002**
	TER	5	53.3	73	0.2716	**0.0001**
	PLA	7	56.7	75	0.2485	**0**
	SVI	6	56.7	58.3	0.0286	0.1255
	CHA	7	43.3	67.7	0.3653	**0.0011**
	PET	8	66.7	71.7	0.0698	0.2929
	PIN	5	50	68.3	0.2738	**0.0085**
A-115	ACR	12	57.7	82.3	0.3023	**0.0009**
	BEL	13	76.7	83.7	0.0863	0.2186
	COL	3	0	61.3	1,000	**0**
	PLA	12	53.3	82.3	0.3564	**0**
	TER	5	28.6	66.8	0.5765	**0.0002**
	PLA	5	56.7	75.3	0.2519	0.0621
	SVI	6	30	64	0.5364	**0**
	CHA	9	40	64	0.3797	**0.0005**
	PET	5	50	58.3	0.1454	**0.0017**
	PIN	6	36.7	57.3	0.3652	0.0219

Inbreeding coefficient (F_is_). In bold, significant P value (α = 0.05) after Bonferroni correction rejects the Hardy-Weinberg equilibrium. p <0.00083.

The COL population was monomorphic, sharing the same allele in most individuals, in the *loci* CA-118 and ATG-9, 56 alleles from 60 have the same fragment size. Population RBR had thirty alleles with the same size (106 bp) for the locus GT-14 and was not found in any other population. COL showed the same phenomenon with A-115 locus, where 28 alleles have 296 bp and can only be found in this population.

### Genetic differentiation

In order to quantify levels of genetic structure among populations the F_st_ index (genetic heterogeneity) was used. The F_st_ mean value was 0.12 indicating moderate genetic structuring, pairwise F_st_ value comparisons between populations ranged from 0.08 to 0.29 and all of them were statistically significant (P < 0.01; Table 4).

**Table 4:** **Lower diagonal shows pairwise F**_**st**_ **estimates**

**Population**	**RBR**	**BEL**	**COL**	**LPL**	**TER**	**PLA**	**SVI**	**CHA**	**PET**	**PIN**
RBR	-									
BEL	0.046	-								
COL	0.236	0.310	-							
LPL	0.136	0.075	0.344	-						
TER	0.052	0.058	0.281	0.146	-					
PLA	0.052	0.033	0.290	0.090	0.045	-				
SVI	0.116	0.087	0.353	0.089	0.137	0.064	-			
CHA	0.100	0.107	0.270	0.105	0.129	0.052	0.052	-		
PET	0.050	0.116	0.263	0.146	0.106	0.067	0.099	0.043	-	
PIN	0.098	0.083	0.293	0.086	0.116	0.035	0.046	0.027	0.050	-

The statistical significance of F_st_ estimates was assessed using 10000 permutations. All comparisons were statistically significant (p < 0.01).

### Genetic distance

**Figure 2:**
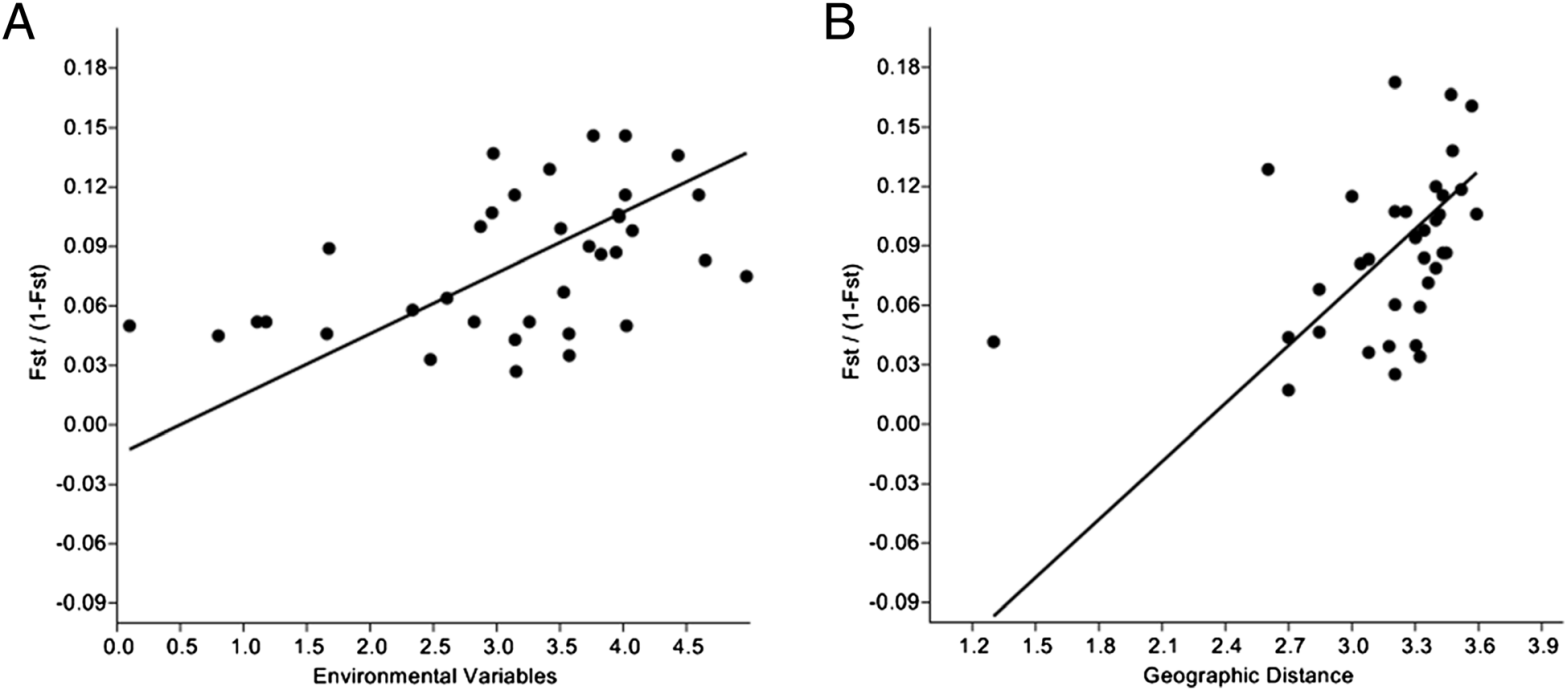
**Fst (Fst/(1-Fst) upon environmental variables and linear distance. A**-Linearized F_st_ values (F_st_/(1-F_st_) upon environmental variables between pairs of populations, showing a significant IBD effect (r = 0.46; p > 0.004) between analyzed populations. **B**-Linearized F_st_ values (F_st_/(1-F_st_) upon linear distance (in km) between pairs of populations, showing a significant IBD effect (r = 0.51; p > 0.001) between analyzed populations. (COL is not shown).

Statistically significant correlation was detected between genetic distance (estimated as F_st_/(1 - F_st_)) and geographic distance between populations (r = 0.51; r^2^ = 0.26; p = 0.001) as well as statistically significant correlation between genetic distance and Environmental Variables (r = 0.46; r^2^ = 0.21; p > 0.004) (Figure 2). The Pairwise multilocus estimated number of migrants (Nm) between populations per generation was 2.60736.

**Figure 3:**
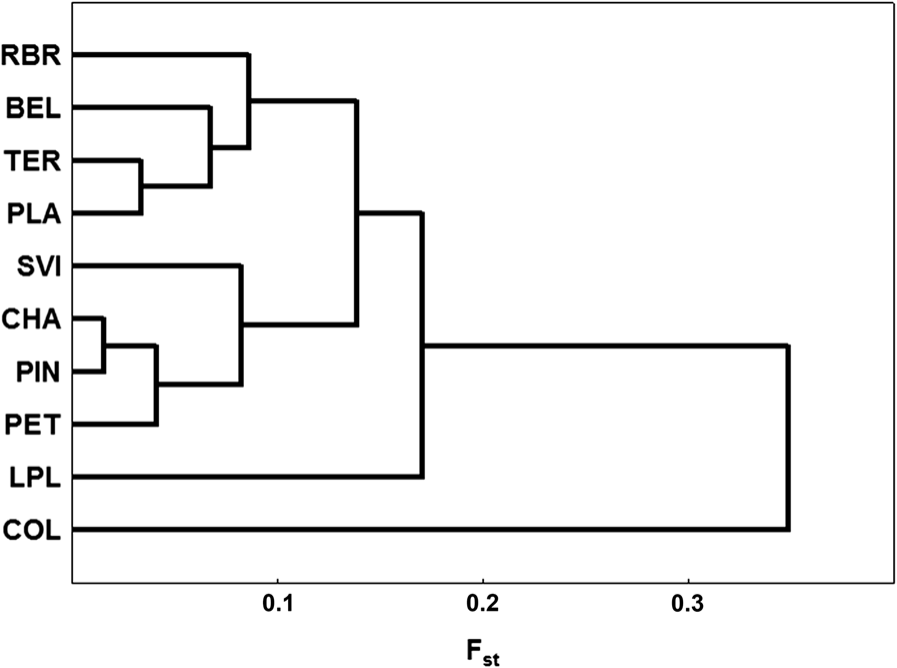
**Genetic distance dendrogram of** ***Culex quinquefasciatus*** **populations.** Statistically significant for all iterations.

The dendrogram of genetic distances based on the allelic variability of the six microsatellite *loci* (GT-108, ATG-09, CA-118, GT-14, GA-12, CA-115) for 10 populations of *Cx. quinquefasciatus* is presented in Figure 3. The dendrogram revealed two clusters, which corresponded geographically to Northern and Southern sampling sites. Population samples LPL (hybrid population) and COL (laboratory colony) were not grouped with any other populations, and the population COL proved to be the most distinct from the other populations, all results were statistically significant.

### Bayesian cluster analyses

**Figure 4:**
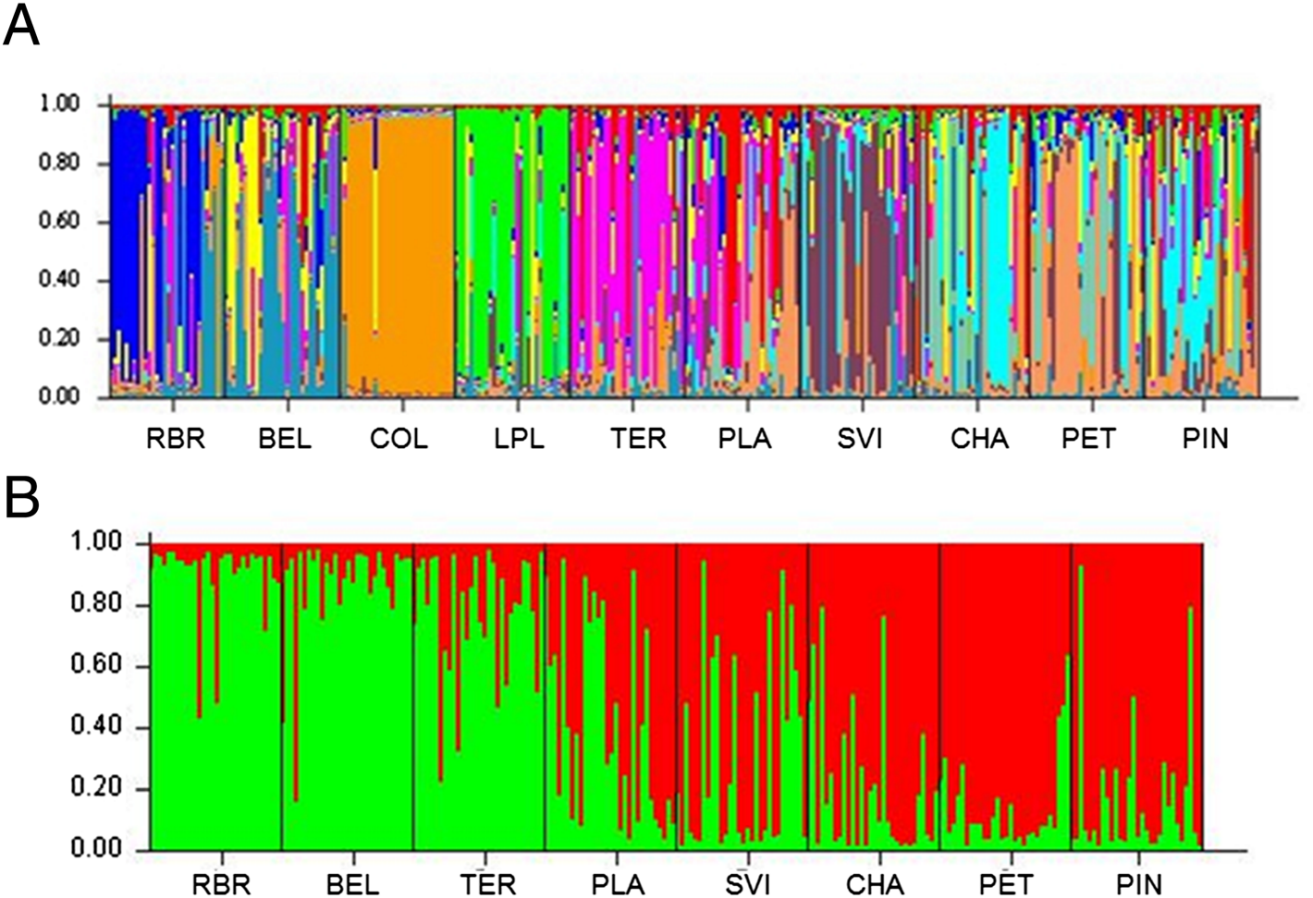
**Multilocus cluster Bayesian analysis of microsatellite genotypes. A**-Multilocus cluster Bayesian analysis of microsatellite genotypes (K = 10)**.** Each of the 300 individuals included in the analysis is represented by a vertical line (each population is composed of 30 mosquitoes) divided into segments of colors that represent the probability of each individual to belong to any of the genetic clusters. 1- RBR, 2- BEL, 3- COL, 4- LPL, 5- TER, 6- PLA, 7- SVI, 8- CHA, 9- PET and 10- PIN**. B**-Multilocus cluster Bayesian analysis of microsatellite genotypes (K values = 2). Each individual is represented by a vertical line and each population is composed of 30 mosquitoes. The populations COL and LPL have not been included in this analysis. Each of the 240 individuals from the remaining populations was represented by a vertical line, divided into segments of colours that represent the probability of each individual to belong to any of the genetic clusters. 1- RBR, 2- BEL, 5- TER, 6- PLA, 7- SVI, 8- CHA, 9- PET and 10- PIN.

The multilocus cluster Bayesian analysis of all 10 population samples together indicate genetic structuration among *Cx. quinquefasciatus*’ populations (delta k = 10) (K value = 10) (Figure 4A). A second multilocus cluster Bayesian analysis was made without COL because it was no longer considered as a sylvatic population (colony was originated in 1980) and LPL was removed because it is actually a hybrid population between *Cx. pipiens* X *Cx. quinquefasciatus* (delta k = 8) (K value = 2) [30] (Figure 4B).

## Discussion

Microsatellite markers were polymorphic and exposed a significant regional differentiation, which is compatible with the population structuration hypothesis for *Cx. quinquefasciatus*. Population structure comprised a clear North–south dichotomy in clustering. Such interpretation is in accordance to Morais *et al.* [30], who first noted that this species varies geographically in the Neotropics using morphometric wing characters [29].

The presence of correlation between genetic and geographic distances suggests that genetic isolation by distance might occur but considering that samples came from different biomes, ecological components are also an influential factor on population structure genetic characteristics as seen in Figure 2. However, our interpretations are yet limited since Brazilian territory was not homogeneously covered, and thus, it may represent several distinct scenarios.

Concerning the northern cluster the geographic and genetic distances did not correlate. For example, TER and PLA although being 2100 km apart, they appeared adjoining in the dendrogram. This discrepancy may be an indirect result of environmental constraints; because both are located in similar ecosystems (transition between semi-arid to rain forest). RBR population is located in a remote region of Brazil where urban mobility is mostly by boat or plane, leading to an isolated scenario, which might have increased the genetic differentiation found in that population.

Located in the southern branch, the SVI population presented lower genetic variability probably because it is located in the southernmost border of geographic distribution (temperature fluctuations may be limitrophe for *Cx. Quinquefasciatus)* [30]. Comparatively, CHA and PIN populations were highly polymorphic, arguably because of an ancestral polymorphism retention, favoured by the fact that environmental constraints are not so restrictive as those of SVI.

Apart from the main dichotomy in the dendrogram, one cannot make deep interpretations regarding the lower clusters up to the present time. Concerning the discrepant samples, COL is a highly inbred and monomorphic laboratory colony and thus appeared to be eccentric; LPL is a hybrid population between *Cx. quinquefasciatus/pipiens* [29] and was genetically distinct from all Brazilian natural populations.

Bayesian analysis confirmed the results obtained by the dendrogram and F_st_ correlation showed significant differences among populations and thus low genic flow. The great distances (>1000 of range in km between populations) and distinct biomes might block migratory flows, if there is gene flow it occurs in a smaller geographic scale.

The deficit of heterozygotes, found in all *loci*, can be explained by a number of non-mutually excludable factors: Population substructure (Wahlund effect), inbreeding and genetic drift [41]. The possibility that some *loci* are under selective pressure, although remote, cannot be discarded. Our interpretation of the results is equivalent to those of da Costa-Ribeiro *et al.* [42].

Taken together, our exploratory findings suggest that *Cx. quinquefasciatus* has a complex population structure and a broad genetic variability. This thought is compatible with the available epidemiological and biological data of the species. *Cx. quinquefasciatus* mosquitoes can be found in urban habitats where there is a great thermal range, manmade selective pressures and several kinds of available breeding sites, features that can lead to temporal variations of clustering and population differentiation [42]. Insects of epidemiological importance might be dispersed, expanding its borders when associated with humans. *Aedes aegypti* mosquitoes can be disseminated by ground transportation as roads and railways are likely to spread dengue virus beyond the normal reach of mosquitoes but are unlikely to spread dengue virus over large areas [42].

Interactions between vector, host and pathogen can change patterns of disease transmissibility resulting in selection, genetic drift and hybridization [43-45]. Parasite and host bond can undergo effects caused by vectors, the most usual effect is the genetic diversity of parasites caused by the variation in the genetic structure of vectors. Parasite strains may be transmitted beyond primary to secondary host populations increasing population numbers of parasites, thus resulting in the decrease of genetic diversity caused by genetic drift or the recurrence of more virulent strains, or the outgrowth of resistance proportioned by the high levels of genetic diversity [46]. *Cx. quinquefasciatus* vector competence is genetically interconnected to *Wuchereria bancrofti* causing significant differentiation among populations [43].

Population genetic analyses can be useful to enlighten demographic and dispersal trends in mosquitoes, leading to uncovering disease patterns and epidemiological changes in transmission dynamics [47,48].

Sunil et al. [49] found that environmental and ecological factors are not the main causes for the genetic differentiation between populations of *Anopheles culicifacies*, an important vector of malaria in Southeast Asia.

Kothera *et al.* [44] suggest that models of disease transmission can be achieved by population genetics and epidemiological data, therefore, improving the efficacy of mosquito control methods. Venktesan & Rasgon [46] state that population dynamics and spread of *Culex tarsalis* mosquitoes may have an association in the invasion of North America by WNV. Fonseca *et al.* [50] showed that mosquitoes from the *Culex pipiens* complex from North America and Europe have distinct epidemiological patterns that can lead to changes in their vectorial capacity, becoming new efficient vectors if introduced into new areas.

## Conclusions

The understanding of population structure of *Cx. quinquefasciatus* mosquitoes, as well as their territorial variations and genetic characteristics might help to predict the introduction of this mosquito into new areas that can lead to disease outbreaks, it could also help with the development of new control strategies. Further studies should aim at better understanding of population and demographical dynamics for smaller geographic scales where there is the possibility of migration and a more intense gene flow.
